# High prevalence of rectal gonorrhoea among men reporting contact with men with gonorrhoea: Implications for epidemiological treatment

**DOI:** 10.1186/s12889-015-1971-3

**Published:** 2015-07-14

**Authors:** Krishneel Dutt, Eric P.F. Chow, Sarah Huffam, Karen Klassen, Christopher K. Fairley, Catriona S Bradshaw, Ian Denham, Marcus Y. Chen

**Affiliations:** Melbourne Sexual Health Centre, Alfred Health, Melbourne, VIC Australia; Central Clinical school, Monash University, Melbourne, VIC Australia; Faculty of Medicine, Melbourne University, Melbourne, VIC Australia

**Keywords:** Gonorrhoea, Men who have sex with men, MSM, Epidemiological treatment, Empiric treatment, Contact

## Abstract

**Background:**

This study aimed to determine the prevalence of gonorrhoea and factors associated with rectal gonorrhoea among men reporting sexual contact with men with gonorrhoea.

**Methods:**

Men who presented to Melbourne Sexual Health Centre reporting sexual contact with a male with gonorrhoea were prospectively identified between March 2011 and December 2013. These men were screened for pharyngeal and rectal gonorrhoea using culture. The prevalence of gonorrhoea among contacts was compared to that among all men who have sex with men (MSM) screened at the clinic over the same period.

**Results:**

Among 363 contacts of gonorrhoea the prevalence of rectal gonorrhoea was 26.4 % (95 % CI: 21.8 %-31.0 %) compared to 3.9 % (95 % CI: 3.7 %-4.2 %) among clinic attendees (*p* < 0.001). The prevalence of pharyngeal gonorrhoea among contacts was 9.4 % (95 % CI: 6.4 %-12.4 %) compared to 2.1 % (95 % CI: 1.9 %-2.4 %) among clinic attendees (*p* < 0.001). Among contacts who reported not always using condoms during receptive anal sex with casual partners, rectal gonorrhoea was cultured in 42.4 % compared with 12.7 % among contacts reporting no receptive anal sex (*p* < 0.001) and 20.2 % among those reporting always using condoms (*p* < 0.001). On multivariate analysis rectal gonorrhoea was associated with inconsistent condom use during receptive anal sex with casual partners (adjusted odds ratio (AOR): 4.16; 95 % CI: 1.87-9.26) and a reported past history of gonorrhoea (AOR: 1.77; 95 % CI: 1.01-3.14).

**Conclusions:**

The high proportion of positive cases of gonorrhoea among contacts in this study supports epidemiological treatment of MSM presenting as contacts of gonorrhoea.

## Background

Gonorrhoea remains one of the most prevalent sexually transmitted infections (STIs) worldwide [1], with substantial prevalence reported among men who have sex with men (MSM) [2]. The reported prevalence of rectal and pharyngeal gonorrhoea among MSM in selected studies has ranged from 3.4 % to 6.9 % [3–7] and 3.9 % to 9.2 % [3–7] respectively. Urethral gonorrhoea typically manifests with purulent urethral discharge while rectal and pharyngeal infections are usually asymptomatic. Rectal gonorrhoea is believed to increase the risk of HIV transmission between men [8]. The continued transmission of gonorrhoea is of public health concern due to increasing antimicrobial resistance to various classes of antibiotics including extended spectrum cephalosporins [9–12].

The public health control of gonorrhoea in MSM has to a large extent hinged on screening and treatment: current guidelines recommend screening MSM for gonorrhoea at least annually with more frequent screening for higher risk men [13,14]. The Australian STI testing guidelines suggest that all MSM be screened for rectal and pharyngeal gonorrhoea, while testing for urethral gonorrhoea is reserved only for men with urethral symptoms [13–15]. Chlamydial screening from the rectum and urethra are also recommended as part of the screening process [13,14]. A number of clinical guidelines recommend that individuals presenting as sexual contacts of partners diagnosed with gonorrhoea be treated empirically for gonorrhoea at the first clinic visit [13,16,17]. However, evidence for this policy in the case of MSM is not available.

This study aimed to provide data on the prevalence of gonorrhoea among men reporting sex with a man infected with gonorrhoea and factors predictive of gonorrhoea infection among such men.

## Methods

This study was conducted at the Melbourne Sexual Health Centre, the main public sexually health clinic in Melbourne, Victoria, Australia. In order to prospectively identify patients presenting as sexual contacts of individuals with gonorrhoea, all patients reporting contact with gonorrhoea were systematically recorded in the electronic medical record from March 2011. Such patients were identified by the clinic’s triage nurse so that epidemiological treatment for gonorrhoea could be offered in all cases. These men will henceforth be referred to as “contacts” of gonorrhoea. Clinical data including sexual histories routinely obtained by computer assisted self-interview (CASI) and the result of laboratory investigations were obtained on all MSM reporting contact with gonorrhoea from March 2011 to December 2013.

Men were considered MSM if they reported a male sex partner within the last year. MSM were routinely asked a series of pre-specified questions regarding their recent sexual behaviours using CASI, including whether they had a regular or casual sexual partners together with questions on condom use for insertive and receptive anal sex. The CASI did not ask about oral sex. MSM were not required to undertake CASI if they had completed it at a previous visit within 3 months. Those who did not undertake CASI were excluded from the study. Also excluded were men who presented to the clinic because they had sexual contact with a gonorrhoea infected male more than once within 4 weeks. These men were excluded as clinic policy was to undertake a test of cure one week after treatment for gonorrhoea.

All asymptomatic MSM attending the clinic were routinely screened for gonorrhoea with a pharyngeal and an anal swab for culture using modified Thayer-Martin medium, as nucleic acid amplification testing for gonorrhoea was not used at the centre over the study duration. Inoculated culture plates were immediately taken to the clinic’s onsite laboratory and intubated at 36° in 5 % carbon dioxide for 48 h. Presumptive *N. gonorrhoeae* colonies were selected and a Gram stain smear prepared. Further testing using oxidase and carbohydrate reactions aided in speciation. Urethral swabs for gonorrhoea culture were only obtained in men who presented with urethral discharge. In addition urine and an anal swab were obtained for chlamydia screening using strand displacement amplification.

The prevalence of contacts in the study who tested positive for pharyngeal, rectal and urethral gonorrhoea was determined. These data were compared with the background prevalence of gonorrhoea among all MSM who attended the clinic over the same time period. Behavioural factors potentially associated with gonorrhoea infection among contacts of gonorrhoea were explored using the Chi-square test for categorical variables. Those variables with a p-value <0.1 were entered into a logistic regression model using SPSS version 21. Ethical approval for this study was granted by the Alfred Hospital Research Ethics Committee (300/13).

## Results

Over the study period there were 363 clinic visits by 346 contacts presenting to the clinic reporting sexual contact with a man with gonorrhoea and who undertook CASI. This included 13 contacts who presented twice and 2 who presented on three occasions at least 4 weeks apart. There were 38 MSM who presented as contacts but were excluded because they did not undertake CASI. During the 363 clinical presentations by contacts, there were 362 pharyngeal swabs, 352 anal swabs and 59 urethral swabs obtained for gonorrhoea culture. Over the same period there were 20,377 clinic visits by 9,108 MSM during which 19,793 pharyngeal, 18,117 anal, and 2,251 urethral swabs were obtained for gonorrhoea culture.

Among the 363 contacts of gonorrhoea, 107 (29.5 %, 95 % confidence interval (CI): 24.8 %-34.2 %) had gonorrhoea isolated at the pharynx (*n* = 34), rectum (*n* = 93) or urethra (*n* = 5). Concurrent infection of the pharynx and rectum occurred in 6 % of contacts. Gonorrhoea was isolated in the urethra and pharynx in one contact, and in the urethra and rectum in two contacts. Of the 15 contacts who had repeat visits, only one had gonorrhoea isolated on both occasions.

Rectal gonorrhoea was isolated in 26.4 % (95 % CI: 21.8 %-31.0 %) of contacts, several fold higher than that among all clinic MSM attendees over the same period: 3.9 % (95 % CI: 3.7 %-4.2 %; *p* < 0.001). Pharyngeal gonorrhoea was isolated in 9.4 % (95 % CI: 6.4 %-12.4 %) of contacts, significantly higher than the 2.1 % (95 % CI: 1.9 %-2.4 %; *p* < 0.001) seen in all clinic MSM attendees over the same time. The prevalence of rectal chlamydia among contacts of gonorrhoea was also significantly higher than among clinic MSM attendees (12.3 % *vs* 6.4 %; *p* < 0.001). Urethral gonorrhoea was isolated in 8.5 % (8/59; 95 % CI: 0.8 %-16.2 %) of contacts tested for urethral gonorrhoea compared to 24.3 % (547/2251; 95 % CI: 22.5 %-26.1 %) among all MSM attendees tested for urethral gonorrhoea over the same period.

The median age among contacts was 28 years (range: 16–60). The sexual relationships and behaviours reported by contacts overall are summarised in Table 1. Among contacts who reported engaging in anal sex, nearly half had casual sex partners only while a fifth had a regular male partner only. The remaining contacts had a regular partner as well as casual sex partners. Over three quarters of contacts with a regular sexual partner did not use condoms all the time during anal sex with their regular partner. Around half of contacts did not use condoms all the time with casual sex partners (Table 1).

**Table 1:** Sexual relationships and behaviours reported by men reporting sexual contact with men with gonorrhoea

Sexual relationships and behaviours	n (%)
Sexual relationships	
No. of men who reported insertive and/or receptive anal sex with a male partner	325
Men who had regular and casual male sex partners^a^	100 (30.7 %)
Men who had a regular sex partner only	71 (21.8 %)
Men who had casual sex partners only	154 (47.4 %)
Condom use with regular sex partners	
No. of men who reported insertive anal sex with a regular sex partner	151
Men who reported not always using condoms during insertive anal sex with a regular partner	118 (78.1 %)
No. of men who reported receptive anal sex with a regular sex partner	159
Men who reported not always using condoms during receptive anal sex with a regular partner	124 (78.0 %)
Condom use with casual sex partners^a^	
No. of men who reported insertive anal sex with a casual sex partner	225
Men who reported not always using condoms during insertive anal sex with casual partners	116 (51.6 %)
No of men who reported receptive anal sex with a casual sex partner	227
Men who reported not always using condoms during receptive anal sex with casual partners	119 (52.4 %)

^a^Relationships and condom use over prior 3 months

Factors associated with rectal gonorrhoea infection among men reporting sex with gonorrhoea infected men are shown in Table 2. In univariate analysis the factors associated with rectal gonorrhoea were: sex overseas; not always using condoms during receptive anal sex with casual male partners; drug use associated with unprotected anal sex; and having unprotected anal sex with a partner whose HIV status was unknown. In multivariate analysis rectal gonorrhoea was significantly associated with not always using condom use during receptive anal sex with casual partners (adjusted odds ratio (AOR): 4.16; 95 % CI: 1.87-9.26) and a self-reported past history of gonorrhoea (AOR: 1.77; 95 % CI: 1.01-3.14).

**Table 2:** Factors associated with rectal gonorrhoea among men reporting contact with men with gonorrhoea

	Number of with positive cultures (%)	Number with negative cultures (%)	Total Number	Odds Ratio (95 % CI)	*p* value	Adjusted Odds Ratio (95 % CI)	*p* value
Age							
≥28	43 (22.9)	145 (77.1)	188	1			
<28	50 (30.5)	114 (69.5)	164	1.48 (0.92 - 2.63)	0.107		
Sex overseas in the past 12 months							
No	52 (22.1)	183 (77.9)	235	1		1	
Yes	32 (33.3)	64 (66.7)	96	1.76 (1.04 – 2.97)	0.035	1.77 (0.98 – 3.18)	0.057
Number of male sex partners in past 3 months							
0-2	29 (20.9)	110 (79.1)	139	1		1	
≥3	63 (30.1)	146 (69.9)	209	1.62 (0.99 – 2.68)	0.056	0.96 (0.49 – 1.89)	0.913
Condom use with regular sex partner during receptive anal sex							
No receptive anal sex	134 (74.0)	47 (26.0)	181	1			
Always used condoms	7 (21.2)	26 (78.8)	33	0.77 (0.31 – 1.89)	0.564		
Did not always use condoms^b^	34 (27.9)	88 (72.1)	122	1.10 (0.66 – 1.85)	0.714		
Condom use with casual sex partner during receptive anal sex in past 3 months							
No receptive sex	13 (12.7)	89 (87.3)	102	1		1	
Always used condoms	21 (20.2)	83 (79.8)	104	1.73 (0.82 – 3.68)	0.153	1.50 (0.65 – 3.44)	0.339
Did not always use condoms^c^	50 (42.4)	68 (57.6)	118	5.03 (2.53 – 10.01)	<0.001	4.16 (1.87 – 9.26)	<0.001
HIV status							
HIV negative or unknown	88 (27.1)	237 (72.9)	325	1			
HIV positive	5 (18.5)	22 (81.5)	27	0.62 (0.26 – 1.67)	0.337		
Past history of gonorrhoea							
No	50 (22.8)	169 (77.2)	219	1		1	
Yes	40 (32.3)	84 (67.7)	124	1.61 (0.99 – 2.63)	0.058	1.77 (1.01 – 3.10)	0.048
Drug use with unprotected anal sex in the past 12 months^a^							
No	26 (27.4)	69 (72.6)	95	1			
Yes	27 (44.3)	34 (55.7)	61	2.11 (1.07 – 4.15)	0.031		
Unprotected anal sex with a partner of unknown HIV status in the past 12 months^a^							
No	24 (25.8)	69 (74.2)	93	1			
Yes	30 (44.8)	37 (55.2)	67	2.33 (1.19 – 4.55)	0.013		

^a^CASI only asked these questions if men were HIV negative and if they reported unprotected anal sex for either insertive or receptive anal sex with casual or regular partners. Because these were only reported by a small proportion of men these were not included in the multivariate analysis

^b^This included 65 men who reported never using condoms for receptive anal sex and 57 reporting sometimes using condoms for receptive anal sex

^c^This included 6 men who reported never using condoms for receptive anal sex and 113 reporting sometimes using condoms for receptive anal sex

Among 118 contacts who reported not always using condoms during receptive anal sex with casual partners (112 men reporting sometimes using condoms and 6 men reporting never using condoms) the prevalence of rectal gonorrhoea was 42.4 % (95 % CI: 33.4 %-51.4 %). This was significantly higher than the 12.7 % seen among contacts who reported no receptive anal sex with casual partners (*p* < 0.001). Among contacts diagnosed with rectal gonorrhoea 15.1 % reported always using condoms for receptive anal sex while 4.3 % reported no receptive anal sex.

## Discussion

In this study of men presenting to a sexual health service reporting sexual contact with a man with gonorrhoea, overall 26.4 % were diagnosed with rectal gonorrhoea by culture. Among the subset of contacts of gonorrhoea who reported either inconsistent or no condom use for receptive anal sex with casual partners, the prevalence of rectal gonorrhoea was higher – 42 %. These data lend empirical support to the recommended practice of providing epidemiological treatment of individuals reporting sexual contact with gonorrhoea, in this case, where the patient and partner are MSM.

As culture has poor sensitivity for detecting gonorrhoea at extragenital sites the true prevalence of pharyngeal and rectal gonorrhoea among men in this study will have been higher than the rates reported here. The sensitivity of culture for the detection of pharyngeal gonorrhoea compared with nucleic acid amplification testing (NAAT) in four studies of MSM was 47 % [18], 40 % [3], 39 % [19] and 60 % [20]. In the same four studies the sensitivity of culture for rectal gonorrhoea compared to NAAT was 56 % [18], 53 % [3], 86 % [19] and 50 % [20], respectively.

When interpreting these data it should be noted that we do not know if reported partners actually had gonorrhoea, and if they did, their site of infection. Nor do we know if contacts in the study were infected by or transmitted infection to their partner. Given most contacts in this study had pharyngeal or rectal infection, it is possible urethral gonorrhoea was common among partners. This would fit with the observed protective effect of condoms during receptive anal sex against rectal gonorrhoea. Because the data were not collected we are unable to comment on the influence of oral sex on the prevalence of pharyngeal gonorrhoea among contacts.

As data on the actual number of sexual acts with infected partners was not available, per act transmission probabilities could not be calculated; however, they are consistent with a relatively high rate of gonorrhoea transmission via anal sex. It remains uncertain whether sex acts other than oral and anal sex are important in the transmission of gonorrhoea between men. Although activities such as fingering and rimming have been proposed [21], firm evidence for these practices remain elusive. That some of the contacts in this study had verified rectal gonorrhoea in the absence of reported receptive anal sex or with reported consistent condom use might suggest alternative modes of acquisition though, alternatively, they may simply reflect inaccurate reporting [22].

## Conclusions

Justification for epidemiological treatment for a curable sexually transmitted infection, that is, treatment of a contact prior to the availability of test results depends on the rate of infection among such contacts, the level of side effects, and the extent to which infected contacts default from treatment because of loss to follow up. The high proportion of positive cases of gonorrhoea among contacts in this study, particularly among those engaging in unprotected receptive anal sex, supports the need for epidemiological treatment for men reporting sexual contact with males with gonorrhoea. Most guidelines currently recommend treating uncomplicated gonorrhoea with a combination of single dose azithromycin and ceftriaxone where in non-allergic patients serious adverse events are rare. Furthermore the study findings emphasise that promotion of condom use for receptive anal sex remains at the cornerstone of reducing transmission of rectal gonorrhoea.

## Abbreviations

AOR: Adjusted odds ratio
CASI: Computer assisted self-interview
CI: Confidence interval
MSM: Men who have sex with men
NAAT: Nucleic acid amplification testing
STIs: Sexually transmitted infection
